# How May Ramucirumab Help Improve Treatment Outcome for Patients with Gastrointestinal Cancers?

**DOI:** 10.3390/cancers13143536

**Published:** 2021-07-15

**Authors:** Ming-Huang Chen, Sheng-Nan Lu, Chien-Hung Chen, Peng-Chan Lin, Jeng-Kai Jiang, Yulia D’yachkova, Mariusz Lukanowski, Rebecca Cheng, Li-Tzong Chen

**Affiliations:** 1 Taipei Veterans General Hospital, Taipei 11217, Taiwan; mhchen9@vghtpe.gov.tw (M.-H.C.); jkjiang@vghtpe.gov.tw (J.-K.J.); 2 Kaohsiung Chang Gung Memorial Hospital, Kaohsiung City 83301, Taiwan; lushna@cgmh.org.tw; 3 Department of Internal Medicine, National Taiwan University Hospital, Douliu 64041, Taiwan; chenhcc@ntu.edu.tw; 4 National Cheng Kung University Hospital, National Cheng Kung University, Tainan 70403, Taiwan; pengchanlin@gmail.com; 5 Statistics, Eli Lilly GmbH, 1030 Vienna, Austria; dyachkova_yulia@lilly.com; 6 Global Medical Affairs, Eli Lilly Denmark, Hovedstaden, 2730 Herlev, Denmark; lukanowski_mariusz@lilly.com; 7 Eli Lilly and Company (Taiwan) Inc., Taipe City 10543, Taiwan; cmh0827@gmail.com; 8 National Institute of Cancer Research, National Health Research Institutes, Tainan 70456, Taiwan; 9 Kaohsiung Medical University Hospital, Kaohsiung Medical University, Kaohsiung City 80756, Taiwan

**Keywords:** gastrointestinal, gastric cancer, colorectal cancer, hepatocellular carcinoma, ramucirumab, angiogenesis

## Abstract

**Simple Summary:**

Malignancies of the gastrointestinal (GI) tract are among the five most common cancers worldwide. Despite significant therapeutic improvements over the last decade, this group of cancers is characterized by high recurrence rates and a dismal prognosis. There is an urgent need for new therapeutic approaches. New blood vessel formation is pivotal to tumor growth. Ramucirumab (Cyramza^®^ (Eli Lilly and Company, IN, USA)) is a monoclonal antibody that specifically binds to and blocks the activation of vascular endothelial growth factor (VEGF) receptor 2. Ramucirumab is approved for a number of GI tract malignancies either as monotherapy or in combination with chemotherapies. Here, we give an overview of the survival and tolerability data for ramucirumab from phase 3 randomized controlled clinical trials in GI cancers including those from important pre-specified patient subgroups and evidence from real clinical practice worldwide. Our aim is to summarize these data to demonstrate how ramucirumab may help improve treatment outcome for patients with GI cancers.

**Abstract:**

GI cancers are characterized by high recurrence rates and a dismal prognosis and there is an urgent need for new therapeutic approaches. This is a narrative review designed to provide a summary of the efficacy as measured by overall survival, progression free survival, and safety data from phase 3 randomized controlled GI clinical trials of ramucirumab including those from important pre-specified patient subgroups and evidence from real clinical practice worldwide. Quality of life (QOL) is discussed where data are available. Our aim was to summarize the efficacy and safety of ramucirumab in the treatment of GI cancers using these existing published data with a view to demonstrating how ramucirumab may help improve treatment outcome for patients with GI cancers. The data indicate that ramucirumab is efficacious, safe, and tolerable across the intent-to-treat patient populations as a whole and across several pre-specified subgroups, even those whose disease is traditionally more difficult to treat. Furthermore, survival outcomes observed in real-world clinical practice demonstrate similar data from phase 3 clinical trials even in patients with complications, suggesting that the benefits of ramucirumab translate in actual clinical practice.

## 1. Introduction

### 1.1. Gastrointestinal (GI) Cancers

Both the incidence of cancer and cancer mortality are growing rapidly worldwide. Malignancies of the gastrointestinal (GI) tract such as the esophagus, pancreas, stomach, colon, rectum, anus, liver, biliary system, and small intestine are among the five most common cancers in both men and women worldwide [1]. GI cancers are overall the most common causes of cancer-related death in men. The multiple vague and non-specific symptoms contribute to the late diagnosis of GI cancers with approximately 50 percent of patients having a disease that extends beyond locoregional confines at the time of presentation [2]. Signs of undernutrition are often observed in patients with GI cancers. Parallel to loss of body weight, cachexia often occurs, and is associated with reduced quality of life (QOL) and prognosis [3]. Comorbidities have a negative prognostic impact on overall survival in patients with cancer [4] by either having a direct effect on survival, being associated with increased toxicity of specific treatments or the use of less optimal or aggressive therapy, thereby reducing a patient’s remaining life expectancy. The majority of GI cancers appear to arise sporadically, with only a small percentage having an apparent hereditary component [5]. Few GI cancers are associated with driver genetic alterations that have a distinct role in the initiation and progression of the cancer.

### 1.2. Targeting Angiogenesis for the Treatment of GI Cancers

Angiogenesis is pivotal to tumor growth—the rate of tumor cell proliferation and metastases is known to be directly related to angiogenic activity [6]. The development, relapse, and spread of tumors depends on new blood vessels that provide the nutrition, growth factors, and oxygen required for continuous tumor growth. The vascular endothelial growth factor (VEGF) family consists of five ligands (placental growth factor [PGF] and VEGF-A, -B, -C, and -D) and three receptors, VEGFR1, VEGFR2, and VEGFR3, which are present on the surface of endothelial cells of blood vessels and lymphatics, respectively [7]. VEGF-A/VEGFR2 -mediated signaling is important in angiogenesis necessary for tumor growth [8] and VEGF-A is a key mediator in the angiogenic switch from an avascular to vascular phenotype without which tumors would not be able to grow bigger than 1–2 mm [8,9].

Known to be involved in multiple angiogenic processes including endothelial cell survival, proliferation, migration, and hyperpermeability, VEGF-A is also known to have nonangiogenic tumor-promoting effects including suppression of the antitumor immune response by inhibiting the maturation of dendritic cells, inhibition of tumor cell apoptosis, the stimulation of metastasis, and increasing the permeability of tumor blood vessels, which reduces the delivery of chemotherapeutic agents to the tumor [10,11]. Furthermore, there are reports of VEGF-mediated signaling having direct, autocrine effects by promoting tumor cell survival, and contributing to tumor invasiveness by carcinoma cells expressing VEGFR-1, VEGFR-2, and neuropilin 1 (NRP1) [12].

Thus, tumor angiogenesis represents a rational target for cancer therapy [13]. By targeting the genetically stable proliferating endothelial rather than tumor cells, drug resistance is less likely. Furthermore, since endothelial cells are in constant contact with the blood (and therefore blood borne agents), the issue of tumor drug delivery is circumvented.

### 1.3. Objective

Our aim was to summarize the efficacy and safety of ramucirumab in the treatment of GI cancers using overall survival (OS), progression-free survival (PFS), and tolerability data for ramucirumab from phase 3 randomized controlled GI clinical trials including those from important pre-specified patient subgroups and evidence from real clinical practice worldwide, with a view to demonstrating how ramucirumab may help improve treatment outcome for patients with GI cancers.

## 2. Materials and Methods

### 2.1. Literature Review and Selection for GI Cancers

#### 2.1.1. Randomized Control Trials (RCTs)and Secondary Analyses

A literature search was conducted in May 2020 with the published scientific literature contained within the PubMed and OVID MEDLINE^®^ (Bethesda, MD, USA), electronic databases. Search terms used were: “advanced gastric cancer”, “advanced colorectal cancer”, “advanced hepatocellular carcinoma (HCC)” and “ramucirumab”. The inclusion criteria were (a) study design—original randomized control trial or secondary analysis; (b) full text availability; (c) articles published in English; and (d) articles published between 2015 to 2020. Exclusion criteria were articles published in a language other than English and commentary manuscripts. Articles were read and assessed for relevance.

#### 2.1.2. Real World Evidence

The targeted literature review was conducted in May 2020 using the PubMed and OVID MEDLINE^®^ (Bethesda, MD, USA), electronic databases for reports on the treatment of advanced gastric cancer and/or GEJ cancer in the real world. Search terms included free text and controlled vocabulary terms such as “ramucirumab + treatment regimens” and “unresectable, locally advanced gastrointestinal cancer” or “metastatic gastrointestinal cancer”. Inclusion criteria included (a) article type—reports, press releases, websites and policy documents, and (b) articles published in English. Exclusion criteria were articles published in a language other than English and commentary manuscripts. These results were supplemented with manual searches to identify further relevant studies.

### 2.2. Statistical Methods

This is a narrative review designed to provide a summary of the efficacy of ramucirumab as measured by OS and PFS and safety data in the existing available literature. QOL data are discussed where data are available. No new analyses were performed.

A table summarizing the overall efficacy endpoints from the phase 3 ramucirumab gastrointestinal clinical trials is provided. In addition, forest plots for patient level data from the ramucirumab phase 3 gastrointestinal clinical trials: RAINBOW, REGARD, RAISE, REACH, and REACH 2 were created to visually display the estimated treatment effects for the endpoints of OS and PFS across different studies and subgroups. They show the estimated hazard ratios (HRs) and corresponding 95% confidence intervals (CI) around the ratio. Since these summaries are based on published analyses conducted with distinct objectives, a variety of model structures were utilized. HR and 95% CI were calculated using a stratified or adjusted Cox models unless indicated with an asterisk (*) where an unadjusted Cox model was used. In some of the reported studies, a treatment-by-subgroup interaction *p*-value was calculated to determine whether the treatment effect was consistent between subgroups. Because the studies were not powered to show the significance in subgroups, the interpretation of significance is not warranted, and should be used as an indication only.

## 3. Results

### 3.1. Ramucirumab

Ramucirumab (Cyramza^®^, (Eli Lilly and Company, IN, USA) LY3009806) is a fully human IgG1 monoclonal antibody that specifically binds to and blocks the activation of VEGF receptor 2 (R2) by its ligands VEGF-A, -C, and -D [14]. In contrast to other agents directed against the VEGFR-2/VEGF axis, ramucirumab binds a specific epitope on the extracellular domain of VEGFR-2, thereby blocking all VEGF-ligand bound downstream signaling from this therapeutically validated target. Preclinical models demonstrated that ramucirumab could potentially selectively bind to and inhibit the human VEGFR-2 with a greater affinity than its natural ligands [15]. The combined effects of high specificity and highly efficacious target inhibition suggested that ramucirumab could potentiate a substantial blockade upon angiogenesis [16].

Ramucirumab demonstrated significant antitumor activity across a range of malignancies in animal models, both as a single agent and in combination with other therapeutics [17]. Furthermore, since nonclinical toxicology studies have suggested that ramucirumab was well tolerated, a phase 1 study commenced treating patients with advanced solid malignancies including those with colorectal and gastric cancers [18]. The apparent disease control rate from this study was 73%, with 30% of patients experiencing either partial response or stable disease for six months or longer, thus suggesting that VEGFR-2 blockade with ramucirumab may be an effective anticancer strategy.

### 3.2. Gastric Cancer

#### 3.2.1. Incidence, Mortality, and Treatment

According to GLOBOCAN 2018 data, gastric cancer is the third leading cause of cancer deaths worldwide, accounting for 8.2% of cancer-related deaths [1]. First-line treatment of advanced or metastatic gastric or gastroesophageal junction (GEJ) adenocarcinoma involves platinum and fluoropyrimidine chemotherapy, with a third agent sometimes added based on human epidermal growth factor receptor 2 (HER2) status [19]. Upon disease progression, second-line chemotherapy with irinotecan, or a taxane, often yields a survival benefit over best supportive care [20,21].

#### 3.2.2. REGARD

REGARD (NCT00917384) was an international multicenter randomized double blind placebo-controlled phase 3 trial designed to assess the safety and efficacy of ramucirumab monotherapy in patients with advanced gastric (aGC) or gastroesophageal junction (GEJ) adenocarcinoma who had disease progression after first-line chemotherapy [22]. Ramucirumab significantly improved OS relative to placebo (5.2 months for patients in the ramucirumab group versus (vs.) 3.8 months for those in the placebo group, hazard ratio [HR] = 0.78, 95% CI [0.60–1.0]; *p* = 0.047) as well as PFS (2.1 months for patients receiving ramucirumab vs. 1.3 months for those receiving placebo, HR = 0.48 [0.38–0.62]; *p* < 0.0001), as seen in Table 1. Well tolerated in this patient population, the rates of adverse events (AE) were similar between the ramucirumab and placebo groups with fatigue, abdominal pain, and decreased appetite the most commonly reported in both arms. Of the adverse events of special interest (AESI), 8% of ramucirumab treated-patients experienced grade ≥3 hypertension relative to 3% in the placebo arm. Grade ≥3 arterial thromboembolic events were slightly more common in the ramucirumab group (1%) than in the placebo group (0) (*p* = 0.55). Although QOL data were available for >95% of patients at baseline, data were scarce at the first scheduled post-baseline assessment at six weeks due to discontinuation of therapy, particularly in the placebo arm. There were no statistical differences between the proportions of patients with improved or stable global QOL scores between treatment arms from those patients who provided data at six weeks [22]. An exploratory analysis demonstrated that time to deterioration (TTD) in the Eastern Cooperative Oncology Group (ECOG) performance status (PS) to a score of 2 or worse was longer in the ramucirumab-treated patient group relative to placebo (median TTD 5.1 months vs. 2.4 months, HR = 0.59 [0.41–0.83], *p* = 0.002) [23]. Alternative definitions of PS deterioration yielded similar HRs [23].

#### 3.2.3. RAINBOW

A second international randomized, placebo-controlled, double-blind, phase 3 trial, RAINBOW, was designed to assess the efficacy of ramucirumab in combination with paclitaxel relative to placebo plus paclitaxel in a similar patient population to that assessed in the REGARD study [24]. Both OS and median PFS were significantly increased in the ramucirumab plus paclitaxel group compared with the placebo and paclitaxel group (median OS 9.6 months [95% CI 8.5–10.8] vs. 7.4 months [95% CI 6.3–8.4], stratified HR 0.81 [95% CI 0.68–0.96]; *p* = 0.02; median PFS 4.4 months [95% CI 4.2–5.3] vs. 2.9 months [2.8–3.0]; stratified HR 0.64, [95% CI 0.54–0.75]; *p* < 0.0001).

The incidence of grades 3 or 4 treatment emergent adverse events (TEAEs) that were more common in the ramucirumab plus paclitaxel group in RAINBOW included neutropenia (grade 3, 22% vs. 16% and grade 4, 19% vs. 3%), leukopenia (grade 3, 16% vs. 6% and grade 4, 2% vs. 1%), abdominal pain (6% vs. 3%), and fatigue (12% vs. 5%) compared to the placebo plus paclitaxel group. Although the incidence of grades 3 or 4 neutropenia was higher in the ramucirumab plus paclitaxel group, the incidence of grade 3 or greater febrile neutropenia was similar in both groups (3% vs. 2%). Grade 3 hypertension also occurred more frequently in the ramucirumab plus paclitaxel group (14% vs. 2%). Other AE of special interest (AESI) that were more common in the ramucirumab plus paclitaxel group included proteinuria (1% vs. 0), and bleeding or hemorrhage (4% vs. <1%). The incidences of grades 4 and 5 AESI were low in both groups, with no grades 4 or 5 hypertension, similar incidence of gastrointestinal hemorrhage (<1% for grade 4 and 5 for both arms), and a higher incidence of gastrointestinal perforation in the ramucirumab plus paclitaxel group than the placebo plus paclitaxel group (<1% vs. 0 for grades 4 and 5). However, the overall higher rate of grades 3 or 4 AEs in the ramucirumab plus paclitaxel group did not result in a higher number of patients discontinuing, or a higher number of deaths, than in the placebo plus paclitaxel group. Although not statistically significant, HRs for TTD in QOL favored ramucirumab plus paclitaxel in 14 of the 15 scales of the European Organization for Research and Treatment of Cancer Quality of Life Questionnaire (EORTC QLQ-C30). TTD in PS to ≥2 also favored ramucirumab plus paclitaxel (HR = 0.80, *p* = 0.0941) and alternate definitions of PS deterioration yielded similar results [42].

##### Subgroup Analysis from RAINBOW and REGARD–the East Asian Subpopulation

Approximately half of the total world gastric cancer cases occur in East Asia and this region also has the highest estimated mortality rates for the malignancy [43]. Subgroup analysis of East Asian patients from the REGARD trial (*n* = 26) indicated that ramucirumab prolonged median OS (6.5 months ramucirumab-treated vs. 4.8 months placebo-treated, HR 0.69, 95% CI [0.27–1.82]) in East Asian patients, which was consistent with the improvement in ramucirumab-treated patients in the study overall [29]. The main limitation of this analysis was the low number of East Asian patients that were enrolled in REGARD. A similar analysis from the RAINBOW trial data (East Asian patients *n* = 223) demonstrated that treatment with ramucirumab and paclitaxel resulted in a median OS 12.1 months relative to 10.5 months for placebo plus paclitaxel (HR: 0.99, 95% CI [0.73–1.3] *p* = 0.929) and significantly improved PFS (median PFS 5.5 vs. 2.8 months, ramucirumab and paclitaxel vs. placebo and paclitaxel, HR 0.63 [0.47–0.83] *p* = 0.001) [32].

In Japan, ramucirumab treatment, as a single agent or in combination with paclitaxel, has been assessed for patients with advanced gastric cancer with or without disease progression after first-line therapy in several real-world clinical practice settings [44,45,46,47,48,49,50,51,52,53,54]. Results appear to be consistent with those from the RAINBOW and REGARD trials. These studies included patients with large amounts of ascites [44], in patients categorized as elderly [46,47,49,52], and in those with liver metastases [55]. Finally, in Korea, patients enrolled in an expanded access program (EAP) received ramucirumab in combination with paclitaxel or with ramucirumab monotherapy [56]. Improved OS and PFS was reported in the combination group relative to the monotherapy group in the Korean EAP cohort with a median OS of 8.6 vs. 6.4 months and PFS of 3.8 vs. 1.8 months [56]. Taken together, ramucirumab, in combination with paclitaxel, has been shown to improve outcomes in the East Asian as well as global population, indicating that it may be an appropriate option for the treatment of advanced GC in East Asia.

##### Subgroup Analysis from RAINBOW and REGARD— Human Epidermal Growth Receptor 2 (HER-2) + GC

HER-2+ gastric cancer is an important disease subset associated with poor outcomes and a more aggressive disease [57]. The heterogeneous HER-2 expression observed in patients with gastric cancer [58,59] may limit the efficacy of HER-2-targeted treatments. Trastuzumab, a monoclonal antibody targeting HER-2 inducing an immune-mediated response that causes internalization and downregulation of HER-2, is an approved first-line treatment for patients with aGC/GEJ adenocarcinoma with HER-2 overexpression. However, of the patients who progress during trastuzumab therapy [60], continuation of trastuzumab treatment in combination with paclitaxel does not prolong survival compared with paclitaxel alone [61]. Trastuzumab deruxtecan (Enhertu^®^, Daiichi Sankyo Company, Limited, NJ, USA), an antibody-drug conjugate consisting of the anti-HER2 antibody bound to a cytotoxic topoisomerase I inhibitor [62], has recently been assessed for patients with locally advanced or metastatic HER-2 positive GC/GEJ adenocarcinoma who had received a prior trastuzumab regimen in the phase 2 DESTINY-Gastric01 study [63]. The results have led to accelerated approval in the U.S., but trastuzumab deruxtecan is not yet approved in all geographies, and a confirmatory trial is underway. Ramucirumab treatment, as assessed by the REGARD and RAINBOW randomized trials included patients that had received prior trastuzumab therapy. Currently, ramucirumab in combination with paclitaxel remains a standard of care for patients who progress while receiving platinum-based chemotherapy, regardless of prior trastuzumab therapy. Although there was no association found between HER-2 positivity and ramucirumab efficacy in the REGARD analysis [31], a retrospective subgroup analyses of RAINBOW data reported longer durations of disease control in ramucirumab plus paclitaxel-treated patients than those with placebo plus paclitaxel, in patients who had previously progressed on trastuzumab-based combination therapy [34]. These results have been supported by a retrospective review of patients diagnosed across two centers in Canada and Italy with reported positive responses that were also durable [64], indicating that ramucirumab in combination with paclitaxel is effective with a manageable safety profile for the treatment of patients with HER-2 + GC/GEJ adenocarcinoma who received first-line therapy with trastuzumab.

##### Subgroup Analysis from RAINBOW and REGARD—Patients with Ascites

A frequent complication of advanced gastric cancer, malignancy-related ascites is thought to develop in conjunction with peritoneal dissemination, in part due to the increased VEGF concentration in the peritoneal fluid and the resultant increased peritoneal capillary permeability [65]. Since potentially critical complications such as infection, ileus, peritonitis, and hydronephrosis can follow ascites, clinicians often proceed cautiously with second-line treatments. Post-hoc, exploratory analyses of RAINBOW patient data were performed to examine whether ascites impacted the efficacy and safety of ramucirumab-paclitaxel treatment [33]. Median OS for the with-ascites subgroup was 5.2 months relative to 8.5 months in the without-ascites subgroup, confirming the prognostic importance of this factor. However, ramucirumab treatment effects on OS did not seem to differ significantly among patients with ascites (OS stratified HR = 0.86, 95% CI [0.64–1.16] *p* = 0.34) vs. those without ascites (OS stratified HR = 0.75, 95% CI [0.59–0.94] *p* = 0.01) and similar results were observed for PFS [33]. Ramucirumab-paclitaxel was found to be favorable even in patients with ascites and was consistent with the overall findings of the RAINBOW trial.

##### Subgroup Analysis from RAINBOW and REGARD—Age Subgroups

**Figure 1:**
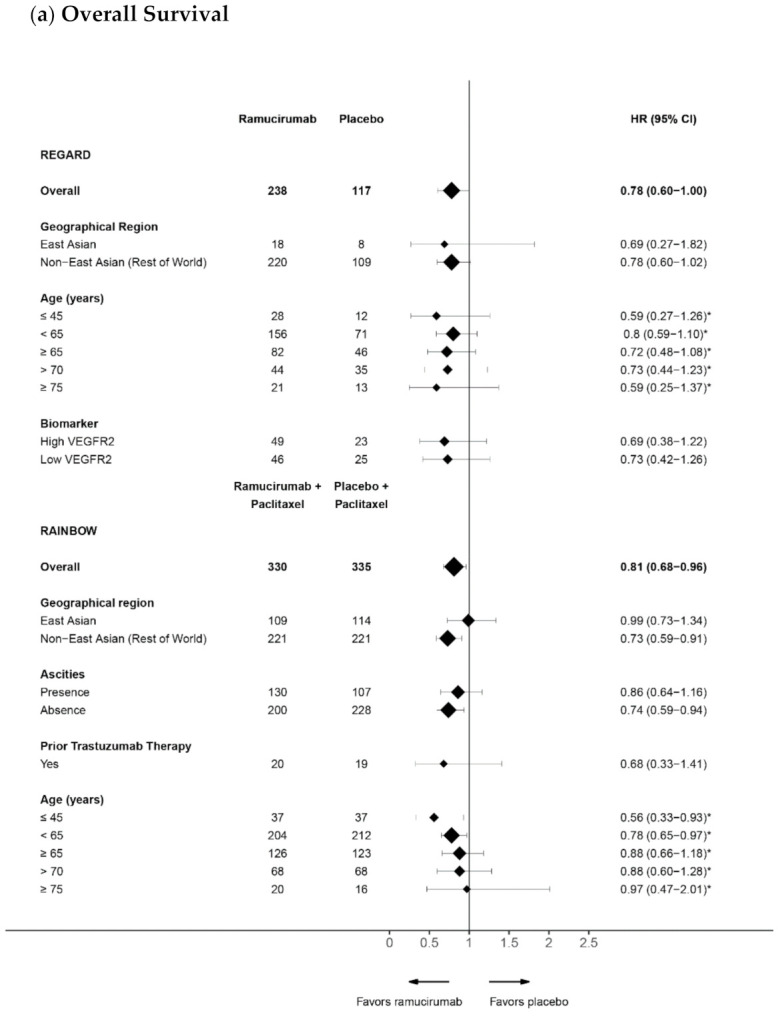

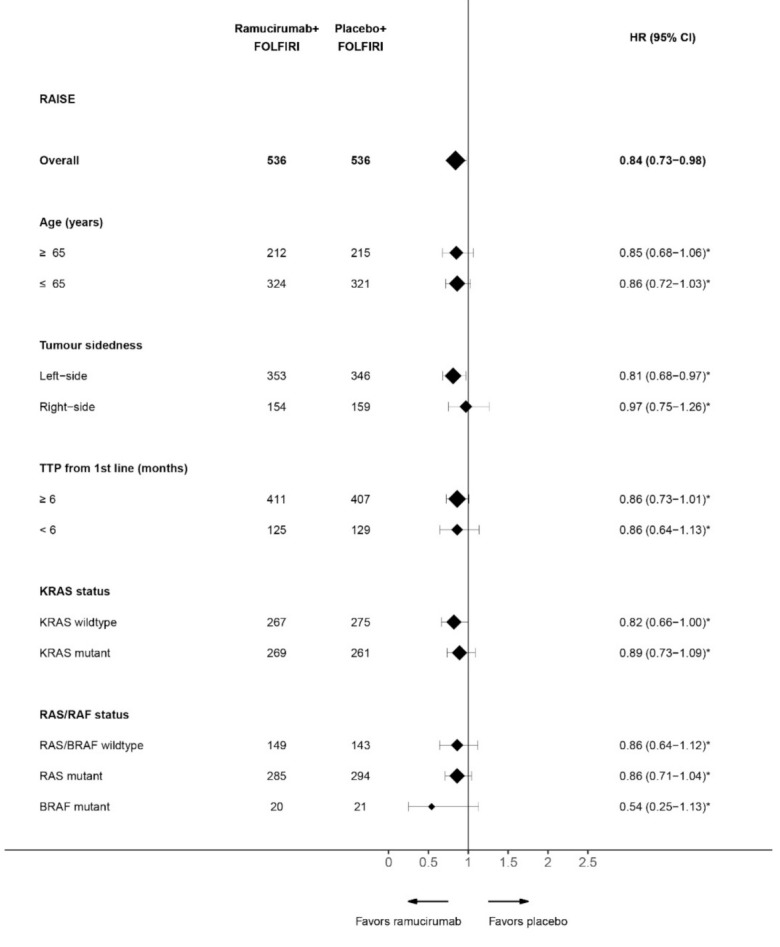

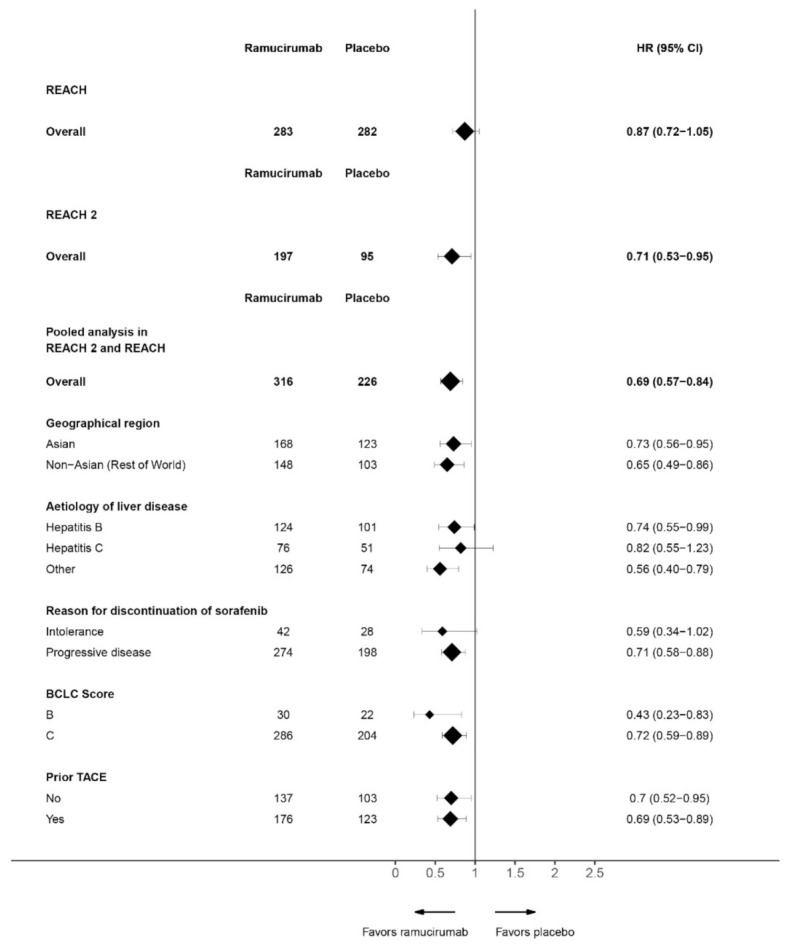

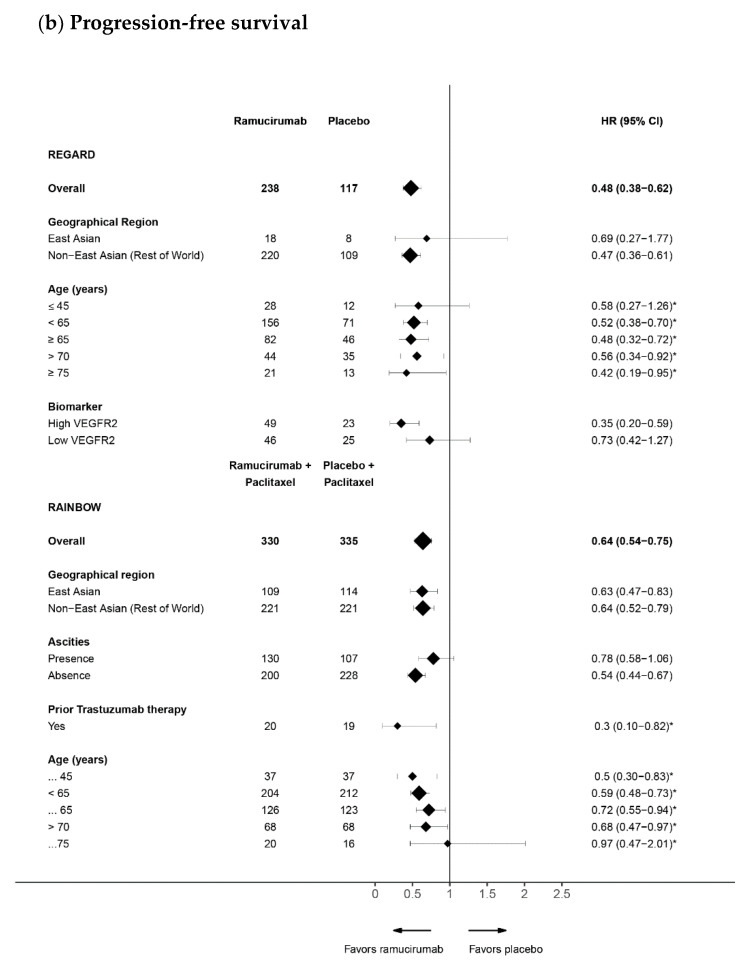

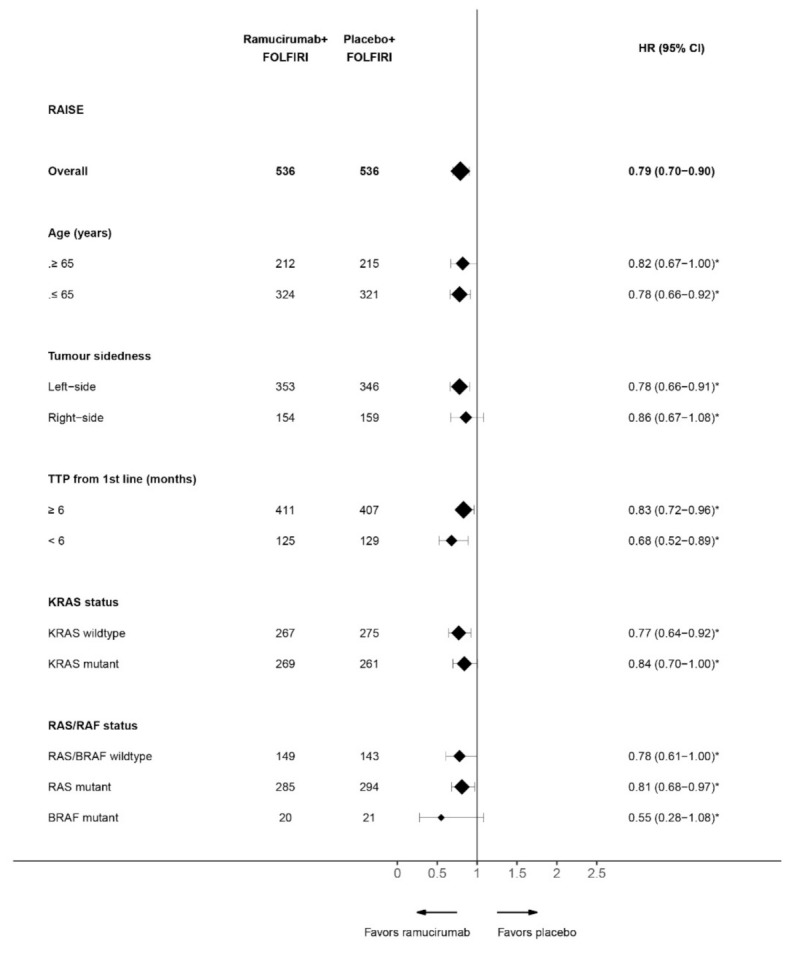

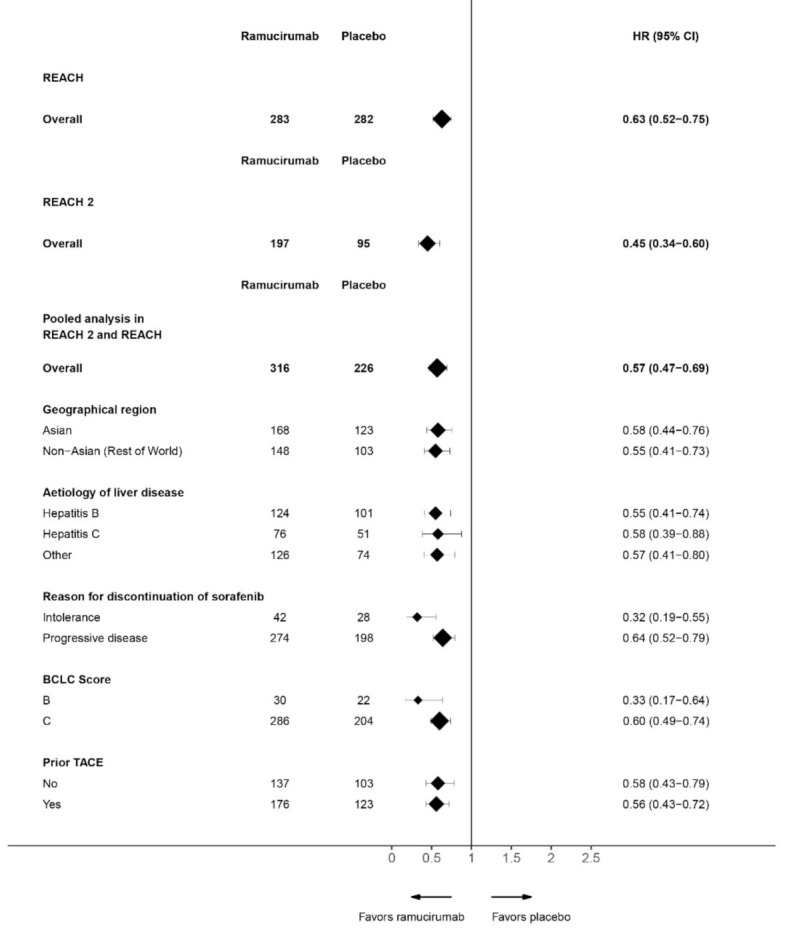
Forest plots for subgroup analyses of (**a**) overall survival (OS) and (**b**) progression-free survival (PFS) in the randomized controlled trails (RCTs) of ramucirumab in gastrointestinal (GI] cancers. Hazard ratios (HR] and 95% confidence intervals (CI) have been calculated using a stratified Cox model unless indicated with an asterisk (*) where an unstratified Cox model was used. The size of the diamond is proportional to the size of the subgroup. Data taken from REGARD [22,29,30,31], RAINBOW [24,32,33,34], RAISE [26,35,36], REACH [27], REACH 2 and pooled analysis [28,37,38,39,40,41].

Age is a clinically important factor when determining treatment for oncology patients. More than 60% of GC cases are diagnosed in patients who are 65 years of age or older and more than one-third of patients are 75 or older [66]. Despite data suggesting that elderly patients with GC are able to tolerate and experience survival benefits to the same extent as younger patients, many oncologists hesitate to recommend systemic chemotherapy to elderly patients, perhaps due to the increased likelihood of comorbidities or poor physical condition with advancing age. The incidence of some AEs such as hypertension, bleeding, and neutropenia are commonly associated with increasing age. Younger patients, although diagnosed relatively infrequently, are more likely to carry genetic abnormalities, and present with aggressive clinicopathological features including poorly differentiated diffuse adenocarcinoma and higher rates of nodal and distant metastases [67]. Although neither RAINBOW nor REGARD were powered for comparison within age groups, a trend for improved survival with ramucirumab was seen across all age subgroups relative to the placebo [30] (Figure 1a). Toxicity profiles were comparable between younger (<65 years, *n* = 416) and older (≥65 years, *n* = 249) patients in RAINBOW, although higher incidences of grade ≥3 neutropenia and leukopenia were noted in patients aged ≥65 years, regardless of treatment arm. Overall, these exploratory analyses support the use of ramucirumab in aGC/GEJ, irrespective of patient age.

#### 3.2.4. Inconclusive Trials

The role of ramucirumab as a first-line treatment in GC/GEJ adenocarcinoma has been investigated in two randomized phase 3 trials. RAINFALL, a randomized, placebo-controlled double-blind phase 3 trial [25], indicated that ramucirumab in combination with first line fluoropyrimidine and cisplatin was well tolerated and significantly improved investigator-assessed PFS. However, the addition of ramucirumab to first-line chemotherapy with cisplatin/capecitabine did not improve OS. RAINSTORM, a phase 2 double-blind trial was conducted in parallel with RAINFALL enrolling East Asian patients with advanced GC/GEJ adenocarcinoma to evaluate the efficacy and safety of ramucirumab in combination with S-1 and oxaliplatin in the first-line setting. The addition of ramucirumab to first-line S-1 plus oxaliplatin treatment did not improve PFS or OS compared with S-1 plus oxaliplatin treatment alone. Taken together, the results of the RAINFALL and RAINSTORM studies suggest that the efficacy of ramucirumab as a second-line therapy may not apply in the first line setting in combination with fluoropyrimidine and platinum chemotherapy.

#### 3.2.5. Ramucirumab for GC in the Real World

The safety and effectiveness of ramucirumab as a treatment option for patients with GC/GEJ adenocarcinoma has also been examined in real world clinical practice in the Western world. For example, in the RAMIS study in Spain [68], the RAMOSS study in Italy [69], along with a few smaller studies [70,71,72,73], all indicated that ramucirumab is well tolerated in real life situations with similar survival outcomes and response rates as seen in the RCTs.

#### 3.2.6. Ramucirumab-FOLFIRI in Second Line

The combination of FOLFIRI plus ramucirumab (FOLFIRI-R] may be an attractive alternative to the paclitaxel ramucirumab combination in the second line. There is a growing number of patients pre-treated with docetaxel in the perioperative or first-line setting [74] whose disease may be taxane resistant. The FOLFIRI-R combination in the second line setting may help to address tumor taxane resistance as well as avoiding the overlapping toxicity profiles that may restrict the reuse of taxanes. Recently, an interim analysis of a multicenter, randomized, investigator initiated, phase 2 trial has demonstrated the efficacy and safety of the combination of FOLFIRI-R as a second-line treatment for aGC/GEJ adenocarcinoma, with a larger survival benefit in comparison to the paclitaxel combination in a post taxane-treated group of patients [75]. These results are supported in clinical practice as evidenced by a retrospective multi-institutional analysis carried out at three academic institutions in the USA [76]. Based on the existing data, irinotecan-based ramucirumab treatment regimens have been added to the National Comprehensive Cancer Network (NCCN] guidelines for aGC as an additional option.

### 3.3. Colorectal Cancer

#### 3.3.1. Incidence, Mortality and Treatment

In 2018, colorectal cancer (CRC) was the third most common cancer and the second leading cause of cancer death worldwide, with over 1.8 million new cases reported [1]. Around half of all patients with CRC develop metastatic disease associated with a poor prognosis and a 5-year survival of just 13%. Standard of care first line treatment for the majority of patients with metastatic colon cancer is the combination of 5-fluorouracil (FU)/leucovorin (LV) with either oxaliplatin (FOLFOX) or irinotecan (FOLFIRI) together with antiangiogenic antibody treatments such as anti-VEGF (bevacizumab) or anti-epidermal growth factors (EGFRs), cetuximab, or panitumumab in patients without RAS mutation [77].

#### 3.3.2. RAISE

In the multicenter, randomized phase 3 clinical trial, RAISE, ramucirumab plus FOLFIRI was compared with the placebo plus FOLFIRI as a second-line treatment for metastatic (m)CRC following first-line combination therapy with bevacizumab, oxaliplatin, and a fluoropyrimidine [26]. This trial was designed to address the question whether second-line treatment with an anti-angiogenesis agent that blocks the VEGF receptor is beneficial after initial antiangiogenic treatment such as bevacizumab. A significant increase in OS was observed in patients in the ramucirumab arm compared with those in the placebo arm (13.3 months [95% CI 12.4–14.5]) for the ramucirumab group versus 11.7 months [10.8–12.7] for the placebo group (stratified HR 0.84, 95% CI [0.73–0.98] *p* = 0.022). Patients treated with ramucirumab/FOLFIRI also demonstrated a significant improvement in PFS versus those treated with placebo/FOLFIRI (5.7 months [95% CI 5.5–6.2] for ramucirumab-treated patients versus 4.5 months [95% CI 4.2–5.4] for placebo-treated patients, HR 0.79, 95% CI [0.70–0.90], log rank *p* = 0.0005). The proportion of patients achieving objective responses and overall disease control was similar in the ramucirumab and placebo groups. In RAISE, TEAEs led to 83% of ramucirumab-treated patients relative to 75% of placebo-treated patients to experience at least one dose modification (reduction, delay, or omission of any study drug), the majority of whom had one or more dose delays. The incidence of dose omissions and dose reductions of ramucirumab or placebo was low and similar in the two groups (8% and 8%, respectively, in the ramucirumab group vs. 6% and 4%, respectively, in the placebo group. Most AEs were manageable with supportive care or dose modification as only 11% of patients in the ramucirumab group and 4% in the placebo group discontinued treatment).

The most common events leading to discontinuation of any treatment component were neutropenia, thrombocytopenia, diarrhea, and stomatitis. A total of 29% of patients in the ramucirumab group versus 13% in the placebo group discontinued at least one component, mostly FOLFIRI components; 4% of patients discontinued ramucirumab and 1% discontinued placebo. Baseline QoL scores were similar between the two treatment arms. The proportions of patients with stable or improved global QoL scores remained largely similar over time. Relative to the placebo arm, transient decreases in most QOL scales were observed in the ramucirumab arm in the first month of therapy, but no differences were observed after two months [78].

##### RAISE Subgroup Analyses—Tumor Mutational Status

Tumor characteristics may affect the efficacy of anticancer drugs and their suitability in certain patient populations. Several subgroup analyses were performed on the RAISE clinical data to identify patient populations who may derive different treatment benefit from ramucirumab. Identified as predictors of resistance to anti-EGFR therapy in patients with mCRC, Kirsten rat sarcoma 2 viral oncogene homolog (KRAS) mutational status (specifically exon 2 mutation status) was a stratification factor in RAISE, with mutant KRAS tumors present in approximately half of the patient sample [35]. Treatment with ramucirumab/FOLFIRI significantly improved OS (*p* = 0.05) and PFS (*p* = 0.06) compared with placebo/FOLFIRI in patients with wild-type tumors, and directional improvements in OS and PFS were observed in the ramucirumab arm amongst patients with mutant KRAS tumors (Figure 1a,b). There was no significant interaction between treatment effect and KRAS mutation status for either OS (interaction *p* = 0.51) or PFS (interaction *p* = 0.53). Other rat sarcoma (RAS) mutations (specifically KRAS exon 3 and 4, and neuroblastoma (N]RAS) and v-raf murine sarcoma viral oncogene homolog B1 (BRAF) mutations may similarly reduce the benefit of anti-EGFR therapies. RAS/BRAF mutation information was available for 85% of the total RAISE sample [36]. As with KRAS analyses, the favorable treatment effects with ramucirumab were comparable between patients with expanded RAS mutations and patients with RAS/RAF wild-type tumors (Figure 1a,b). Treatment-by-mutation status interaction tests indicated that treatment effect did not differ significantly among the three mutation status subgroups (RAS/BRAF wild-type, RAS mutant or BRAF mutant) for either OS (interaction *p* = 0.52) or PFS (interaction *p* = 0.67). Overall, ramucirumab was effective versus placebo regardless of KRAS or RAS mutational status.

##### RAISE Subgroup Analyses—Primary Tumor Location

Primary tumor location (i.e., left or right colon) has been linked to differences in clinical and biological characteristics between patients and, like mutational status, may be predictive of anti-EGFR treatment response [79]. Tumor sidedness was known in 94% of the RAISE intention-to-treat sample. Although ramucirumab was more efficacious in patients with left-sided CRC than in those with right-sided disease (Figure 1a,b), the results from non-significant interaction tests indicated that sidedness was not clearly associated with the OS and PFS benefits of ramucirumab therapy (OS interaction *p* = 0.276; PFS interaction *p* = 0.578) [36].

##### RAISE Subgroup Analyses—Age

Advancing age, often resulting in a greater chance of physiological limitations or co-existing illnesses, is known to be a poor prognostic factor for patients with mCRC. Post-hoc analysis of the RAISE study data found that ramucirumab/FOLFIRI demonstrated similar improvement versus placebo/FOLFIRI in both patients aged <65 and those aged ≥65 years (Figure 1a), and improvements in PFS versus placebo observed with ramucirumab in both older (*p* = 0.05) and younger patients (ns) (Figure 1b) [35]. The treatment effect did not differ between the two subgroups (treatment-by-subgroup interactio*n* = 0.95 for OS and 0.70 for PFS).

##### RAISE Subgroup Analyses–Progression Status

Unlike the RAISE study, some clinical trials excluded patients with rapid progression or who were intolerable to first-line bevacizumab. In the RAISE study, there was no significant interaction between first-line time to progression (TTP) status and treatment effect for either OS or PFS (OS interaction *p* = 0.94; PFS interaction *p* = 0.11). Patients who had progressed in ≥6 months showed survival improvement (Figure 1a) as well as significant improvement in PFS (Figure 1b) [35]. Ramucirumab treatment was also efficacious in the 24% of patients who progressed on first-line therapy in <6 months (Figure 1a,b).

##### RAISE Subgroup Analyses—TEAEs

The incidence of all grades as well as grade ≥3 TEAEs was comparatively consistent across patient tumor KRAS status and the first-line TTP subgroups [35,36]. Neutropenia, thrombocytopenia, stomatitis, epistaxis, and hypertension occurred more frequently amongst patients treated with ramucirumab and FOLFIRI relative to placebo and FOLFIRI regardless of KRAS status or first-line TTP. Some TEAEs such as decreased appetite and fatigue, occur more frequently with increasing age. Despite these differences, older ramucirumab-treated patients in RAISE did not experience these events more frequently relative to those treated with placebo [35].

### 3.4. Hepatocellular Carcinoma (HCC)

#### 3.4.1. Incidence, Mortality, and Treatment

Considered to be the most common primary cancer of the liver, hepatocellular carcinoma (HCC) is a highly vascular tumor. Antiangiogenic agents play a crucial role in the management of advanced HCC. Sorafenib, an orally administered multikinase inhibitor with activity against the RAS/RAF kinases affecting cell proliferation and angiogenesis, was first globally approved for advanced HCC in 2007. In 2018, lenvatinib, was proved to be non-inferior to sorafenib for OS and subsequently received approvals from the U.S. Food and Drug Administration (FDA) and European Medicines Agency (EMA).

Immune checkpoint inhibitors (ICIs) have revolutionized treatment for many kinds of cancers including HCC. Indeed, the combination of the programmed death ligand 1 (PD-L1) immune checkpoint inhibitor atezolizumab and the monoclonal anti-VEGF antibody bevacizumab is considered standard of care for unresectable HCC following the analysis of the IMbrave150 study and subsequent FDA approval for this indication [80,81]. However, despite being associated with 15–20% response rates in phase 3 studies of single-agent treatment in first- and second-line settings [82,83], no single agent PD-1 inhibitors such as nivolumab or pembrolizumab have thus far significantly improved OS as monotherapy for patients with HCC in the first line setting.

In the second line setting, regorafenib, another multikinase inhibitor, was the first drug to be approved for HCC following failure to respond to sorafenib. Cabozantinib, an orally bioavailable inhibitor of tyrosine kinases including tyrosine-protein kinase Met (MET), Axl receptor tyrosine kinase (AXL), Ret receptor tyrosine kinase (RET), FMS-like receptor tyrosine kinase-3 (FLT3), and VEGF receptors, has also been approved for HCC patients who have been previously treated with sorafenib. Nivolumab and pembrolizumab as well as the combination of nivolumab plus ipilimumab (an anti-cytotoxic T-lymphocyte–associated protein 4 [CTLA-4] antibody) have been granted accelerated approval by the FDA for sorafenib-pre-treated patients [84,85,86].

#### 3.4.2. REACH 2

Patients with HCC that have high alpha-fetoprotein (AFP) serum levels have been shown to have significantly higher VEGF tissue expression and micro vessel density [87]. In vitro studies have indicated crosstalk between the AFP and VEGF signaling cascades [87,88]. The pivotal phase 3 trial REACH-2 evaluated ramucirumab versus placebo in patients with advanced HCC (with disease progression on or intolerance to sorafenib) and elevated baseline AFP levels (≥400 ng/mL) [28].

The REACH-2 trial results demonstrated that ramucirumab increased OS relative to placebo treatment (8.5 months vs. 7.3 months for the placebo group (HR of 0.71 [95% CI: 0.53–0.95]; *p* = 0.02). Median PFS was also significantly longer in the ramucirumab group than the placebo group (2.8 months [95% CI 2.8–4.1] vs. 1.6 months [1.5–2.7]; HR 0.45, 95% CI [0.34–0.60]; *p* < 0.0001). Overall, the drug was very well tolerated and hypertension and hyponatremia being the only grade 3 or worse TEAEs noted in 5% or more of patients and at higher frequencies in the ramucirumab group relative to the placebo group. Conversely, increased aspartate aminotransferase concentrations were noted more frequently in the placebo group (5% vs. 3%, placebo vs. ramucirumab). Treatment discontinuation due to TEAEs occurred in 11% vs. 3% in the ramucirumab group relative to the placebo group.

#### 3.4.3. REACH

The efficacy and safety of ramucirumab was previously assessed in the phase 3 RCT, REACH [27]. Although in this trial second-line treatment with ramucirumab did not show an improvement in OS for patients with advanced HCC when compared with placebo in an unselected population, investigators were able to identify a pre-planned subgroup—patients with elevated AFP values (AFP ≥400 ng/mL)—that benefited from ramucirumab treatment. In this subgroup, median OS was improved in the ramucirumab group when compared to the placebo (7.8 months [95% CI 5.8–9.3] versus 4.2 months [3.7–4.8]). Median PFS for patients with AFP values equal or higher than 400 ng/mL treated with ramucirumab was 2.7 months [95% CI 1.5–2.8] versus 1.5 months [1.4–2.1] for the placebo group, HR 0.70, [0.53–0.92]. The interaction between the treatment effect of ramucirumab on survival and baseline AFP concentration was evaluated using a Cox model with baseline AFP fitted as a continuous variable. Furthermore, ramucirumab significantly reduced deterioration in QoL as assessed by FACT-Hepatobiliary Symptom Index-8 (FHSI-8] in this subgroup relative to the placebo (*p* = 0.0381) [27]. Overall results suggested that ramucirumab had an increased efficacy and caused an improvement in QoL in those patients with high levels of baseline AFP and led to the development of the REACH-2 study.

#### 3.4.4. REACH and REACH-2 Pooled Analyses

Because both REACH and REACH-2 were global trials evaluating the same treatment regimen and had similar study eligibility and protocol procedures, individual patient data (stratified by study] from REACH (AFP ≥400 ng/mL) and REACH-2 were pooled, thereby providing a substantially larger patient population for subgroup analyses [28]. This enabled more precise estimation of the treatment effect and greater statistical power for subgroup analyses. The combined population comprised 542 patients with baseline AFP concentrations of at least 400 ng/mL, 316 of whom were assigned to ramucirumab: 226 to placebo. In the pooled analysis of efficacy, median OS was significantly improved in the ramucirumab relative to placebo group (8.1 months [95% CI 6.9–9.3] vs. 5.0 [4.3–6.1]; HR 0.69, 95% CI [0.57–0.84]; *p* = 0.0002), consistent with the individual HRs for OS in patients in both studies who had AFP concentrations of 400 ng/mL or higher. PFS in the pooled analysis was also consistent with those in each study. The same rate and type of TEAEs noted in REACH 2 were noted in the pooled population [28]. These AEs are likely on-target effects from VEGFR-2 inhibition.

With a markedly different toxicity profile from the kinase inhibitors, REACH-2 did not exclude those patients who discontinued sorafenib in the first line due to intolerance. Of those patients, ramucirumab improved treatment outcomes vs. placebo (OS 10.2 months vs. 6.7 months HR 0.59 [95% CI 0.34, 1.02] and PFS 4.4 vs. 1.4 HR 0.32 [0.19, 0.55]) [28]. Similarly, in patients who discontinued sorafenib due to progression, ramucirumab treated patients showed survival benefits relative to the placebo—OS 8.0 vs. 4.7, HR 0.71 [95% CI 0.59, 0.87] and PFS 2.7 vs. 1.6 months, HR 0.64 [0.52, 0.79] (Figure 1a,b). Ramucirumab therefore appears to fulfil the need for a second-line treatment for patients who do not tolerate or whose disease progresses on first-line therapy. A further differentiating factor is that ramucirumab does not seem to cause hand-foot skin reaction (HFSR), unlike that caused by the multikinase inhibitors. Ramucirumab is a viable option for patients with elevated AFP levels who failed first-line therapy because of significant HFSR.

##### REACH and REACH-2 Pooled Subgroup Analyses—Age

The risk of HCC generally increases with advancing age. However, HCC usually presents in younger patients in Eastern Asia and Africa, and relatively later in Japan and Western countries, due in part to differences in etiology across these regions [89]. Treatment of elderly patients with HCC remains an unresolved clinical challenge, with increasing unmet need, especially for those ≥70 years of age who often have comorbidities and poor prognosis. The effects of ramucirumab in relation to age were examined in a post-hoc analysis of the pooled patient data from REACH and REACH-2. Ramucirumab showed a survival benefit across age subgroups (Figure 1a,b). Deterioration of quality of life was also delayed and ramucirumab demonstrated an acceptable safety profile including in patients ≥75 years of age. These data support the use of ramucirumab in advanced HCC with elevated AFP irrespective of age [90].

##### REACH and REACH-2 Pooled Subgroup Analyses—South Asian Subpopulation

Ethnicity is known to influence the efficacy, tolerability, and safety of anticancer treatments [91,92]. Real-world research on treatment patterns in Korea has highlighted the continuing high mortality in HCC, especially among the elevated AFP group, underlying a need for new treatments that can lengthen survival [90] Data were pooled in patients with elevated AFP from the REACH-2 and REACH trials, and an individual patient data meta-analysis was performed in the Asian (*n* = 291) and non-Asian (*n* = 251) patient subgroups [37]. Median OS was significantly longer in the ramucirumab arm relative to the placebo arm for both Asian patients and non-Asian patients (8.1 vs. 4.8 months, stratified HR 0.73 [95% CI 0.56–0.95], *p* = 0.0189) for Asian and (8.0 vs. 5.2 months, stratified HR 0.65 [0.49–0.86], *p* = 0.0028] for non-Asian patients (Figure 1a,b). The most common grade ≥3 TEAE differed between subgroups with hypertension (7.7%), decreased appetite (1.2%), and ascites (1.2%) reported in the ramucirumab arm for Asian patients whilst hypertension (16.9%), ascites (8.8%), asthenia (4.7%), and fatigue (5.4%) were reported for non-Asian patients. This post-hoc subgroup analysis demonstrates that ramucirumab is likely to be beneficial in Asian patients with advanced HCC and elevated AFP level who are intolerant to, or have progressed on, sorafenib.

##### REACH and REACH-2 Pooled Subgroup Analyses—BCLC Stage

Intermediate-stage HCC, as defined as Barcelona Clinic Liver Cancer (BCLC) Stage B, is a heterogeneous disease in terms of liver function and tumor load. An exploratory analysis of outcomes by the BCLC stage was performed with pooled data across both REACH-2 and REACH trials [40]. A consistent treatment benefit for ramucirumab versus placebo was observed across staging in both evaluated trials (median OS Stage B: 13.7 versus 8.2 months, ramucirumab to placebo, HR 0.43 [95% CI 0.23–0.83]) and stage C: 7.7 versus 4.8 months, HR 0.72 [0.59–0.89] (Figure 1a,b).

##### REACH and REACH-2 Pooled Subgroup Analyses—Liver Disease Etiology

HCC commonly occurs as a result of chronic liver disease secondary to viral hepatitis B (HBV) or C (HCV), or other causes (alcohol use being the most common in developed countries), resulting in diverse disease etiologies [93]. Factors contributing to prognosis in HCC are complex, and the contribution of the different etiologies to overall HCC prognosis is unclear. An exploratory analysis was conducted to investigate the efficacy and safety of ramucirumab in advanced HCC patients with elevated AFP from REACH-2 and REACH by liver disease etiology [38]. A consistent treatment benefit for ramucirumab versus placebo was observed across etiology subgroups (OS interaction *p*-value = 0.29, PFS interaction *p*-value = 0.38) and ramucirumab was well tolerated with a similar safety profile in all etiology subgroups (Figure 1a,b).

#### 3.4.5. REACH-2 Open Label Expansion

Emergence of novel therapeutic options means that sorafenib is no longer the only option for first-line treatment for advanced HCC. A limitation of both REACH and REACH-2 was that they did not include patients who received first-line systemic treatment other than sorafenib, since it was the only treatment associated with an OS benefit when the trials were designed. Currently, a global open-label expansion cohort of REACH-2 is ongoing, initiated to study ramucirumab in patients with advanced HCC and baseline AFP ≥400 ng/mL following a non-sorafenib based systemic therapy (NCT02435433) [94]. Approximately 44 patients will be enrolled to receive ramucirumab 8 mg/kg administered intravenously once every 14 days. As of 31 January 2020, 24 patients were enrolled. Interim analysis demonstrated that the most common prior systemic therapies were lenvatinib, monotherapy PD-1, or PD-L1 inhibitor, PD-1 inhibitor plus lenvatinib, and atezolizumab plus bevacizumab. Grade ≥3 TEAEs were reported in 58% of patients, of those, 17% were deemed by the investigator to be related to treatment. Grade ≥3 TEAEs reported in ≥10% of patients were hypertension (*n* = 4; 17%), proteinuria (*n* = 3; 13%), and pneumonia (*n* = 3; 13%). No deaths due to AEs occurred on therapy or within 30 days of treatment discontinuation. With a median follow-up of 6.5 months (range: 1.2–20.7), the median PFS was 5.5 months (18 events; 95% CI 1.3–7.5). The overall response rate (ORR) was 16.7% (95% CI: 1.8–31.6). Reporting of median OS is immature with only ten events recorded.

### 3.5. Unanswered Questions

#### 3.5.1. Angiogenesis Biomarkers

The treatment landscape for GI cancers is still evolving [95]. Biomarkers play an important role in the detection and management of cancer. In GI cancers, there is increasing interest in the development and validation of biomarkers according to tumor type. Prognostic biomarkers enable identification of patients with a more aggressive tumor evolution, whilst predictive biomarkers permit the identification of patients with a higher probability of responding to a specific treatment.

##### Serum AFP in HCC

Serum AFP is widely used in clinical practice for diagnosis, pretreatment prognosis, predicting survival after transarterial chemoembolization, and tumor response to therapy, as it is considered to continuously reflect HCC tumor activity and viable burden [96]. The evidence from the REACH-2/REACH trials and ensuing pooled data analysis demonstrated, for the first time, that baseline AFP levels are also an important predictive biomarker to select those patients with HCC who are set to benefit most from ramucirumab treatment. Elevated serum AFP has been correlated with high VEGFR expression and increased angiogenesis [87]. There is evidence suggesting that AFP expression may be associated with more angiogenic tumors and could denote a particular subclass of HCC [97]. A recent analysis of the molecular profiles of patients with HCC (*n* = 520) from two independent cohorts, confirmed the aberrant tumor overexpression of AFP in patients with serum concentrations >400 ng/mL (AFP-high tumors) [98]. AFP-high tumors were characterized by poor differentiation, enriched progenitor features, and enhanced proliferation, characteristics that are consistent with the prognostic capacity of AFP as well as the increased proportion of AFP high tumors observed in patients with disease progression. Furthermore, it was suggested that the significant activation of VEGF signaling displayed by AFP-high tumors could provide the rationale for the efficacy of ramucirumab in this subpopulation of HCC patients.

##### Other Biomarkers—GC

HCC notwithstanding, there has been a paucity of predictive biomarkers indicating subpopulations of patients with other GI cancers that may derive the greatest benefit from specific antiangiogenic treatments including ramucirumab. Given that elevated levels of some VEGF ligands and receptors have been associated with shorter survival [99,100,101], the candidate biomarkers in serum (soluble (s) VEGFR1, 3; VEGF-C, -D) and tumor biopsy (VEGFR2] were explored for their prognostic and potential predictive value for ramucirumab efficacy [31]. A relationship exists between HER-2 overexpression and VEGF upregulation in breast cancer [102,103]. In gastric cancer, it is thought that HER-2 overexpression through VEGF upregulation might support angiogenesis and tumor growth [104]. Although the exploratory analyses using the cohort of patients from REGARD with available tissue and serum specimens failed to identify a candidate biomarker(s] that would facilitate selection of patients more likely to benefit from ramucirumab, the data did indicate that patients with high VEGFR2 expression may benefit to a greater degree from ramucirumab treatment than those with a low VEGFR2 expression profile [31]. Recently, a retrospective analysis using high-throughput RNA sequencing profiles has demonstrated that three genes are differentially expressed in tumors from patients with gastric cancer whose disease responded to ramucirumab treatment [105], indicating that gene expression analyses may be used to personalize the prescription of ramucirumab in the future.

##### Other Biomarkers—CRC

In colorectal cancer, the RAISE trial design included a prospective and comprehensive biomarker program. One of the planned study end points was to identify predictive biomarkers for ramucirumab efficacy in second line mCRC with mandatory collection of plasma and tumor tissue [26]. Following randomization to treatments, patients were randomly assigned for the biomarker program, to marker exploratory (ME) and marker confirmatory (MC) groups [106]. Evaluation of the combined ME + MC populations found that the median OS was improved in the ramucirumab + FOLFIRI arm compared with placebo + FOLFIRI in the high VEGF-D subgroup whilst a decrease of 0.5 month was observed in the low VEGF-D subgroup. PFS results were consistent with the OS data. VEGF-D was identified as a potential predictive biomarker for ramucirumab efficacy in second line mCRC that warrants further evaluation.

#### 3.5.2. Ramucirumab and Immunotherapy

ICIs targeting the PD-L1–PD-1 axis show durable activity in a subset of patients with cancer [107,108] although, for the majority of patients across a variety of tumor types, treatment with ICIs will not achieve disease control. Mechanisms of resistance to checkpoint inhibitor therapy likely include the inhibitory effects of the tumor microenvironment. Antiangiogenic therapies targeting the VEGF signaling axis have been shown to increase trafficking of T cells into tumors and reduce immunosuppressive cytokines and regulatory T cells [109,110], thus potentially helping to overcome resistance to checkpoint inhibitors.

Clinical studies with antiangiogenic drugs in combination with checkpoint inhibitors have shown enhanced antigen-specific T-cell migration, antitumor activity, and a favorable toxicity profile [111]. The NivoRam study, a phase 1/2 trial (NCT02999295) that evaluated the safety and tolerability of addition of ramucirumab to nivolumab in patients with advanced GC as second-line therapy [112], demonstrated promising efficacy with no new safety signals. Ramucirumab has also been combined with pembrolizumab for patients with previously treated advanced solid tumors including GC or GEJ [113]. Again, a manageable safety profile was reported with favorable antitumor activity in patients with all three cancer types. The efficacy of the combined effects of ramucirumab and another anti-PD-L1, durvalumab, in a variety of cancer types including HCC and GC/GEJ was evaluated in a phase 1b study [114]. The combination generated no unexpected toxicities and antitumor activity was especially evident for patients with high PD-L1 expressing tumors. Currently, a phase 3 study of sintilimab (a PD-1 antagonist) and ramucirumab as first-line treatment for advanced gastric or GEJ adenocarcinoma is underway (NCT04675983).

Thus, dual inhibition of the VEGF–VEGFR2 and PD-1–PD-L1 pathways in patients with previously treated advanced or metastatic cancer appears to be a promising treatment strategy. Combining an antiangiogenic drug and a checkpoint inhibitor may potentially be further enhanced by the addition of chemotherapy. A phase 1/2 trial to determine the recommended dose and for evaluating the efficacy, safety, and predictive biomarkers of the combination regimen of paclitaxel, ramucirumab, and nivolumab in patients with advanced GC as second-line therapy (UMIN000025947) is currently underway. Preliminary results suggest that the combination is efficacious and durable with a manageable safety profile [115].

## 4. Conclusions

Ramucirumab was first approved by the U.S. FDA in 2014 for the treatment of advanced or metastatic gastric or gastroesophageal junction (GEJ) adenocarcinoma as a monotherapy. Since then, it has been approved by the FDA and EMA for the treatment of multiple solid tumors in the advanced setting including aGC/GEJ adenocarcinoma in combination with paclitaxel, colorectal cancer in combination with 5-fluorouracil plus leucovorin plus irinotecan (FOLFIRI), and hepatocellular cancer as monotherapy. A recent retrospective observational study using patient-level data from the Flatiron Health’s electronic record oncology database of over 200 U.S.-based clinicians demonstrated that over one-quarter of patients diagnosed with advanced/metastatic GC/GEJC had no record of receiving systematic therapy. Of the 75% of patients who did receive therapy, as many as a quarter of patients with advanced/metastatic GC/GEJC receive ramucirumab-based therapy regimen in the second-line setting [116].

Benefit versus risk must be evaluated carefully by clinicians and patients when making decisions concerning cancer treatments. Ramucirumab, like other VEGF/VEGFR-axis targeted treatments, is associated with hematologic and cardiovascular toxicities. Here, we detail the results of the phase 2 and phase 3 RCTs supporting the role of ramucirumab across GI cancers. The data indicate that ramucirumab is efficacious, safe, and tolerable across the intent-to-treat patient populations as whole and across several pre-specified subgroups, even those whose disease is traditionally more difficult to treat. These groups include those patients with ascites, those whose disease is progressing very rapidly, and those with high AFP levels. Moreover, survival outcomes observed in clinical practice demonstrate similar data from phase 3 clinical trials even in patients with complications such as ascites, neuropathy, and for those patients that require a taxane-free regimen. This suggests that the benefits of ramucirumab translate in actual clinical practice.

## Figures and Tables

**Table 1:** Summary of survival data from phase 3 clinical trials investigating the use of ramucirumab treatment in patients with gastrointestinal (GI) cancers.

Study Name	Study Design	Treatment Arms	N	Patient Population	Findings
Phase 3 randomized controlled trials (RCTs) in Gastric Cancer					
REGARD [22]	Randomized, dou- ble-blind, placebo- controlled, Phase 3 trial of ramu- cirumab or pla- cebo plus best supportive care	Placebo + best supportive care vs. ramu- cirumab + best supportive care	355	Patients with advanced gastric or gastro- esophageal junction adenocarcinoma and disease progression after first-line plati- num-containing or fluoropyrimidine-containing chemother- apy	Median overall survival (OS) Ramucirumab—5.2 months (interquartile range [IQR] 2.3–9.9) Placebo—3.8 months (1.7–7.1) OS hazard ratio (HR]: 0.776, 95% confidence interval (CI) 0.603– 0.998; *p* = 0.047 Median progression free survival (PFS) Ramucirumab—2.1 months (IQR 1.3–4.2) Placebo—1.3 months (1.1–2.1) PFS HR: 0.483, 95% CI 0.376–0.620; *p* < 0.0001
RAINBOW [24]	Randomized, dou- ble-blind, placebo- controlled, Phase 3 trial of ramu- cirumab + paclitaxel or pla- cebo + paclitaxel	Ramucirumab + paclitaxel or placebo + paclitaxel	665	Patients with advanced gastric or gastro- esophageal junction adenocarcinoma and disease pro- gression on or within 4 months after first- line chemotherapy (platinum plus fluoropyrimidine with or without an an- thracycline)	Median OS: Ramucirumab + paclitaxel - 9.6 months (95% CI 8.5–10.8) Placebo + paclitaxel - 7.4 months (95% CI 6.3–8.4) OS HR: 0.807, 95% CI 0.678–0.962; *p* = 0.017 Median PFS: Ramucirumab + paclitaxel—4.4 months (95% CI 4.2–5.3) Placebo + paclitaxel 2.9 months (2.8–3.0) PFS HR: 0.635, 95% CI 0.536–0.752; *p* < 0.0001
RAINFALL [25]	Randomized, dou- ble-blind, placebo- controlled, Phase 3 trial of cisplatin + capecitabine + ramucirumab or cisplatin + capecit- abine + placebo	cisplatin + capecitabine + ramucirumab or cisplatin + capecitabine + placebo	645	Patients with metastatic, HER2-negative gastric or gastro-esophageal junction adenocarcinoma, an Eastern Cooperative Oncology Group (ECOG] performance sta- tus of 0 or 1, and adequate organ function	Median OS Ramucirumab—11.2 months (IQR 9.9–11.9) Placebo—10.7 months (9.5–11.9) OS HR: 0.962, 95% CI 0.801–1.156, *p* = 0.6757 Median PFS Ramucirumab—5.7 months (IQR 5.5–6.5) Placebo—5.4 months (4.5–5.7) PFS HR: 0.753, 95% CI 0.607–0.935, *p* = 0.0106
**Phase 3 RCT in Colorectal Cancer**					
RAISE [26]	Randomized, dou- ble-blind, Phase 3 trial of ramu- cirumab + FOLFIRI or pla- cebo + FOLFIRI	Ramucirumab + FOLFIRI or placebo + FOLFIRI	1072	patients with colorectal cancer and disease progression during or after first-line ther- apy with bevacizumab, oxaliplatin, and a fluoropyrimidine	Median 0 S Ramucirumab + FOLFIRI—13.3 months (95% CI 12.4–14.5) Placebo + FOLFIRI—11.7 months (10.8–12.7) OS HR: 0.844, 95% CI 0.730–0.976; log rank *p* = 0.0219 Median PFS Ramucirumab + FOLFIRI—5.7 months (95% CI 5.5–6.2) Placebo + FOLFIRI - 4.5 months (4.2–5.4) PFS HR: 0.793, 95% CI 0.697–0.903; log rank *p* = 0.0005
**Phase 3 RCTs in Hepatocellular Carcinoma**					
REACH [27]	Randomized, pla- cebo-controlled, double-blind trial of ramucirumab + best supportive care or placebo+ best supportive care	Placebo + best supportive care vs. ramu- cirumab + best supportive care	565	Patients with hepatocellular carcinoma with BCLC stage C or B disease, Child -Pugh class A liver disease, ECOG performance statuses of 0 or 1, that was re- fractory, or not amenable to locoregional therapy	Median 0 S Ramucirumab—9.2 months (95% CI 8.1–10.6) Placebo—7.6 months (6.0–9.3) OS HR: 0.87, 95% CI 0.72–1.05; log rank *p* = 0.14 Median PFS Ramucirumab—2.8 months (95% CI 2.7–3.9) Placebo—2.1 months (95% CI 1.6–2.7) PFS HR: 0.63, 95% CI 0.52–0.75; log rank *p* < 0.0001
REACH 2 [28]	Randomized, pla- cebo-controlled, double-blind trial of ramucirumab + best supportive care or placebo+ best supportive care	Placebo + best supportive care vs. ramu- cirumab + best supportive care	292	Patient with histologically or cytologically confirmed hepatocellular carcinoma, or di- agnosed cirrhosis, and hepatocellular carci- noma, Barcelona Clinic Liver Cancer stage B or C disease, Child-Pugh class A liver disease, ECOG performance statuses of 0 or 1, α-fetopro- tein concentrations of 400 ng/mL or greater, and had previously received first-line sorafenib	Median 0S Ramucirumab—8.5 months (95% CI 7.0–10.6) Placebo—7.3 months (95% CI 5.4–9.1) OS HR: HR 0.710, 95% CI 0.531–0.949; log rank *p* = 0.199 Median PFS Ramucirumab—2.8 months (95% CI 2.8–4.1) Placebo—1.6 months (1.5–2.7) PFS HR: 0.452, 95% CI 0.339–0.603; *p* < 0.0001
